# Comparison of Total Intravenous Anaesthesia Using Propofol With or Without Sufentanil in Laparoscopic Cholecystectomies

**Published:** 2009-08

**Authors:** M Subrahmanyam, B SreeLakshmi

**Affiliations:** 1 Head of department, Department of Anaesthesiology, Global Hospitals, Lakdi-ka-pool, Hyderabad-500004, A.P., India; 2 DNB student, Department of Anaesthesiology, Global Hospitals, Lakdi-ka-pool, Hyderabad-500004, A.P., India

## Abstract

**Summary:**

Sufentanil is an excellent adjuvant in total intravenous anaesthesia (TIVA). The present study evaluates effectiveness of different concentrations of Sufentanil mixed in propofol for TIVA in laparoscopic cholecystectomy. Sixty adult patients of ASA physical status I or II (randomly divided into 3 groups of twenty each) undergoing elective laparoscopic cholecystectomy were included in this randomised control study. At induction, patients in all groups received i.v. bolus of Sufentanil 1 μg kg^−1^ and continuous infusion of 100 μg kg^−1^ min^−1^. Anaesthesia was maintained with propofol infusion titrated in a range of 75 to 125 μg kg^−1^ min^−1^. Groups S1 and S2 received propofol with Sufentanil added at 1 μg ml^−1^ and 2 μg ml^−1^ concentrations respectively, while group Preceived propofol without Sufentanil. Additional Sufentanil boluses (10 μg) were given to patients in all groups when there was an increase in the heart rate by more than 20 beats per minute or mean arterial pressure by more than 15% above baseline. Perioperative haemodynamic parameters, recovery times and postoperative analgesia were compared across the three groups of patients. Haemodynamic parameters (heart rate, systolic and diastolic blood pressures) were not significantly different across the three groups of patients in the perioperative period. Fewer Group S2 patients required additional Sufentanil boluses to maintain adequate depth of anaesthesia compared to other two groups. Group S2 patients had better post-operative analgesia (*p*=0.01) but prolonged recovery time (*p*=0.001) compared to the other two groups. Sufentanil mixed with propofol provides better haemodynamic stability in laparoscopic cholecystectomies, with lesser requirement for additional Sufentanil boluses, and good postoperative analgesia.

## Introduction

Total intravenous anaesthesia (TIVA) is an evolved concept of general anaesthesia, which obviates the need for volatile anaesthetics. Propofol, a sedative-hypnotic agent with excellent recovery characteristics at the end of infusion and additional anti-emetic property, has become the drug of choice for TIVA. Newer synthetic opioids (fentanyl congeners) provide excellent analgesia and hence are popular adjuvants in TIVA. Sufentanil has been combined with propofol in TIVA for various types of surgeries due to its advantages like synergistic action with propofol, rapid induction, less cardiovascular and respiratory depression, and rapid smooth recovery profile.^1^‐^3^ Additionally, early postoperative analgesia with intraoperative use of Sufentanil has been shown in previous studies to be better than that with fentanyl.^1^ These properties can make Sufentanil an excellent adjuvant to propofol in TIVA for upper abdominal laparoscopic surgeries where the intraoperative haemodynamic fluctuations due to pneumoperitoneum and changes in patient position are better addressed. Sufentanil-propofol TIVA provides clearheaded recovery of consciousness at emergence compared to inhalational anaesthesia and good postoperative analgesia, thus making it a useful combination for conducting these surgeries on a day care basis.^4^‐^6^ However, sufentanil's efficacy as an adjuvant to propofol in TIVA is yet to be addressed in laparoscopic cholecystectomies.

The present study seeks to assess the applicability of sufentanil, harnessing the advantages outlined in earlier studies, in TIVA for laparoscopic cholecystectomy. We hypothesized that continuous infusion of sufentanil and propofol will suppress the haemodynamic response to intubation and surgical stimulation; provide perioperative haemodynamic stability with rapid recovery and excellent postoperative analgesia in patients undergoing laparoscopic cholecystectomies. This hypothesis was tested among three groups of patients undergoing laparoscopic cholecystectomy, referred henceforth as Group S1, Group S2, and Group P. We used two different concentrations of sufentanil added to propofol to determine which combination had maximum benefits with least side effects. Groups S1 and S2 received propofol with sufentanil added at 1 μg ml^−1^ and 2 μg m1^−1^, respectively. Group P received plain propofol without sufentanil. This group was intended to serve as control group.

## Methods

This prospective randomized double blind study was conducted during the years 2006-07 after approval from the institutional ethics committee and written informed consent from the patients. Sixty adult patients (18 to 65 years) of ASA physical status I or II with Mallampati scores 1 and 2; scheduled to undergo elective laparoscopic cholecystectomy were included in the study. The study exclusion criteria were: Body weight more than 90 kg, history of hypertension on medications, history of coronary artery disease with or without percutaneous intervention on medications, history of long tern analgesic use, history of psychiatric disorders, patients with severe hepatic orrenal dysfunction (evident from previous medical records and present investigations) and patients in whom NSAIDS are contraindicated.

Patients who fulfilled the inclusion criteria during the pre-anaesthetic check up were randomly assigned into three groups of twenty each with the help of computer-generated table of random numbers. The operation theatre pharmacist assigned the concentration of sufentanil to be added to the propofol infusion for each group. Solutions of propofol containing different concentrations of sufentanil or no sufentanil were prepared in 50 ml syringes by the operation theatre pharmacist as per the randomization chart for each patient, immediately prior to induction. The intervention allocation was masked from the anaesthesiohgists conducting the study, the patients, and the nurses monitoring the patients in the post-anaesthesia care unit and subsequently in the ward.

Before start of anaesthesia, an intravenous access was secured on each patient with an 18 gauge intravenous catheter for fluid and drugadministration. Pie-induction measurement of heart rate (HR), systolic blood pressure (SBP), diastolic blood pressure (DBP), mean arterial pressure (MAP) and peripheral oxygen saturation (SpO_2_) from the anaesthesia monitor was taken as the baseline measurement. Monitoring was continued throughout the period of anaesthesia and included electrocardiography, pulse oximetry, non invasive arterial pressure and capnography. Patients were pre-oxygenated for 3 minutes with 100% O_2_ by facemask. Anaesthesia was induced with slow i.v. injection of sufentanil 1 μg kg^−1^ and continuous infusion of propofol 100 μg kg^−1^ min^−1^. Loss of response to verbal commands was taken as the end point of induction following which intermediate-acting neuromuscular blocking agent; vecuronium 0.1 mg kg^−1^ was given. Trachea was incubated after 3 minutes of mask ventilation and lungs were mechanically ventilated with O_2_-Air mixture and end-tidal CO_2_ concentration (EtCO_2_) was maintained between 30-40 mm Hg. HR, SBP, DBP, and SpO_2_ were recorded 1, 3 and 5 minutes post-induction.

HR, SBP, DBP, SpO_2_ and EtCO_2_ were monitored throughout the intraoperative period and recorded every 15 minutes in the observation sheet. All patients received propofol infusion titrated to the clinical situation in a range of 75 to 125 μg kg^−1^ min^−1^. Hypotension, defined as systolic blood pressure below 80 mm Hg or mean arterial pressure below 60 mm Hg for more than 5 minutes, was treated by reducing propofol infusion by 10μg kg^−1^ min^−1^, but within the range of 75 to 125 μg kg^−1^ min^−1^. Additional intravenous fluids were given as deemed appropriate. Response was reassessed at 5 minute intervals and the above measures continued until stabilization of blood pressure. Hypertension, defined as systolic blood pressure above 150 mm Hg or mean arterial pressure above 95 mm Hg for more than 5 minutes, was treated by giving additional sufentanil (10 μg) boluses. Sufentanil boluses (10 μg) were also given to patients in all groups when there was an increase in the heart rate by more than 20 beats per minute or mean arterial pressure by more than 15% indicating lightening of anaesthesia. Response was re-assessed at 5 minute intervals and the above measures repeated until stabilization. Neuromuscular paralysis was maintained with timely top up doses of vecuronium. Ten minutes before the anticipated end of surgery (at the start of skin suturing), the infusion was stopped. Total volume of propofol given by infusion for each patient was recorded. Total amount of sufentanil and the number of additional boluses of sufentanil given for each patient was recorded.

Residual neuromuscular blockade was reversed with neostigmine 2.5 mg and glycopyrrolate 0.5mg and trachea was extubated at the end of surgery following usual extubation criteria. The HR, SBP, DBP and SpO_2_ were recorded 1 and 5 minutes post-extubation. All patients received slow i.v. diclofenac 75mg added to 100m1 of normal saline at the end of surgery. Recovery time defined as time from stopping of propofol infusion to complete return of consciousness as assessed by the ability of the patient to open eyes and tell his/her name on questioning, and to maintain adequate spontaneous breathing (respiratory rate above 12 breaths per minute), was recorded. Duration of anaesthesia was recorded in minutes for all the patients.

Patients were shifted to the post-anaesthesia care unit where HR, SBP, DBP, RR and SpO_2_ were recorded every 15 minutes for 2 hours. All patients were given supplemental oxygen with face mask. Postoperatively all patients received oral diclofenac 50mg three times daily. Postoperative pain was assessed for 24 hours by 10-cm visual analogue scale (VAS) on which 0 cm represents no pain and 10 cm represents worst imaginable pain.^6^ In post-anaesthesia care unit or in ward, patients with VAS score above 3cm received rescue analgesic, tramadol 100mg intramuscularly. Number of patients requiring rescue analgesic and the time to first rescue analgesic requirement for each patient, in first 24 hours postoperatively was recorded.

## Statistical analysis

Statistical analyses were conducted on measured clinical data using the Statistics Tool Box of MATLAB software.^7^ Power analysis was conducted *a posterior* and found to be more than 90% for comparing differences between the various parameters of interest.

The statistical means of continuous variables across the three groups (age in years, weight in kilograms and duration of anaesthesia in minutes) were analyzed by one-way analysis of variance (ANOVA). Chi-square (χ^2^) test was used to test differences between the groups for categorical variables (sex and ASA physical status). The generalized linear model repeated measures procedure was used when repeated measurements were made on the same subject, as in the case of haemodynamic measurements over the perioperative period. Haemodynamic parameters (HR, SBP and DBP) were compared during the perioperative period at different time intervals – pre-induction or baseline, post-induction, intraoperative, post-extubation and postoperative, using a Bonferroni adjusted generalized linear model repeated measures procedure. The model includes age, sex, weight, duration of anaesthesia, volume of propofol and sufentanil, and baseline (pre-induction) haemodynamic variables as covariates, and adjusted for potential interactions between covariates.

The total volume of propofol and total amount of sufentanil consumed by the three groups was compared using one-way ANOVA and Fisher tests. The recovery time from anaesthesia was compared between the groups using a one-way ANOVA. The time to first rescue analgesia and the number of times rescue analgesia was given were compared using ANOVA and a χ^2^ test. A *p* value <0.05 was considered statistically significant.

## Results

No patient dropped out after enrolment and none of the potentially eligible patients met the exclusion criteria. Patients in the three groups were comparable as regards age, sex, ASA physical status, and duration of anaesthesia (Table 1). Weight was significantly different between the three groups (*p*=0.04). The mean weight was lower in the Group S2 compared to S1 and P.

**Table 1:** Patients' characteristics, ASA physical status and duration of anaesthesia. Values are numbers or mean ± SD

	Group P	Group S1	Group S2	*p* value
	(n=20)	(n=20)	(n=20)	
Age (yr)	35±11	42±11	38±10	0.11
Weight (kg)	67±8	65±10	60±9	0.04*
Sex, F/M	9/11	12/8	12/8	0.55
ASA physicalstatus, I/II	19/1	18/2	18/2	0.80
Duration of anaesthesia (min)	99±21	95±31	98±17	0.81

* Statistically significant, *p* value <0.05

***Haemodynamic parameters*** The three groups were similar in the baseline or pre-induction haemodynamic variables (Table 2). There was no statistically significant difference across the three groups in systolic and diastolic blood pressures (SBP and DBP) in all the time periods studied. There was no statistically significant difference across the three groups in heart rate (HR)in all but two time periods studied: post-extubation and postoperative intervals (Table 2). However this difference was not clinically significant.

**Table 2:** Haemodynamic parameters HR(bpm), SBP(mmHg), and DBP(mmHg) at various time periods. Values are mean ± SD for groups and *p* values for comparison between groups

	Group P	Group S1	Group S2	P Vs S1	P Vs S2	S1 Vs S2
**Pre-induction**						
HR	85±15	84±11	79±11	0.81	0.16	0.16
SBP	132±10	130±18	132±15	0.67	1.00	0.70
DBP	79±10	81±10	81±10	0.53	0.53	1.00
**Post Induction**						
HR	72±2	68±2	66±2	0.43	0.14	1.00
SBP	101±3	97±3	99±3	0.98	1.00	1.00
DBP	58±2	57±2	55±2	1.00	1.00	1.00
**Intraoperative**						
HR	70±2	73±2	71±2	0.86	1.00	1.00
SBP	112±3	112±3	111±2	1.00	1.00	1.00
DBP	70±2	69±2	65±2	1.00	0.39	0.59
**Post Extubation**						
HR	79±3	89±2	79±2	0.03*	1.00	0.02*
SBP	128±3	126±2	126±2	1.00	1.00	1.00
DBP	77±2	78±2	74±2	1.00	1.00	0.61
**Postoperative**						
HR	69±2	76±2	76±2	0.01*	0.03*	1.00
SBP	119±2	121±2	126±2	1.00	0.20	0.55
DBP	76±2	75±2	76±2	1.00	1.00	1.00

Generalized linear model repeated measures procedure, Bonferroni adjusted. The model includes age, sex, weight, duration of anesthesia, total consumption of propofol and sufentanil, and baseline (pre-induction) hemodynamic variables as covariates, and adjusted for potential interactions between covariates.

* A *p* value <0.05 was considered as significant.

***Consumption of propofol and sufentanil*** Patients in Group S2 (mean volume 56.80 ± 14.62 ml) required less propofol, though not statistically significant, compared with Group P (mean volume 65.50 ± 15.68 ml) and Group S1 (mean volume 58.50 ±20.42 ml) (Table 3). The amount of sufentanil at induction (μg) was highest for Group P compared to other two groups as the body weight was highest in this group. The total consumption of sufentanil was significantly high in Group S2 (184.15±38.30 μg) compared to Group S1 (136.3±27.13 μg) and Group P (81.75±17.79 μg), as expected from the study design.

**Table 3:** Total consumption of propofol and sufentanil among all groups (Values are mean ± SD)

	Group P	Group S1	Group S2	P value	F-value
	(n=20)	(n=20)	(n=20)	(ANOVA)	
Volume of propofol consumed (ml)	65.5±15.68	58.50±20.42	56.80±14.62	0.24	1.455
Amount of sufentanil at induction(mcg)	74.25±1.5	71.3±9.55	65.55±12.22	0.05	3.151
Amount of sufentanil given in infusion with propofol (mcg)	0	58.5±20.42	113.6±29.25	<0.001*	152.18
Amount of sufentanil given as intraoperative boluses*	13.63±5.04	11.81±6.03	14.28±5.34	0.61	0.51
Number of patients who received intraoperative sufentanil boluses	11	11	7	0.05	3.15
Total amount of sufentanil consumed	81.75±17.79	136.3±27.13	184.15±38.30	<0.001*	62.51

* n=11 in P, n=11 in S1 and n=7 in S2,

* Statistically significant, *p* value <0.05

***Requirement of additional boluses of sufentanil*** A total of 13 patients in Group S2 did not require any additional sufentanil to maintain an adequate depth of anaesthesia compared to 9 patients in the other two groups (Table 4). The number of patients requiring additional sufentanil boluses intraoperatively in Groups P, S1 and S2, was 11, 11 and 7, respectively (Table 3). However, the total amount of sufentanil given as boluses was not significantly different among those who required them (Table 3).

**Table 4:** Distribution of the number of patients who required additional intraoperative sufentanil boluses in the three groups

			Number of intraoperative sufentanil boluses			
			0	1	2	3
**Group**	**P**	No. of patients	9	7	4	0
		Frequency percent	45.0%	35.0%	20.0%	0.0%
	**S1**	No. of patients	9	10	0	1
		Frequency percent	45.0%	50.0%	0.0%	5.0%
	**S2**	No. of patients	13	4	3	0
		Frequency percent	65.0%	20.0%	15.0%	0.0%
Total		No. of patients	31	21	7	1
		Frequency percent	51.7%	35.0%	11.7%	1.7%

***Recovery times*** Anaesthesia recovery time (Table 5) was longer for Group S2 (21 ± 9 minutes) compared to S1 (14 ± 6 minutes) and P (14 ± 5 minutes). The difference in the mean recovery times across all the groups has a statistical significance of *p* =0.001.

**Table 5:** Anaesthesia recovery time(Values are mean ± SD) and rescue analgesia in postoperative period

	Group P	Group S1	Group S2	P value
	(n=20)	(n=20)	(n=20)	(test)
Anaesthesia recovery time (min)	14±5	14±6	21±9	0.001*
				(ANOVA)
Number of patients requiring rescue analgesic (VAS≥3)	11	7	2	0.01
				(χ^2^)

* Statistically significant, *p* value <0.05

***Rescue analgesic requirement*** Postoperatively, the number of patients with VAS scores above 3 requiring rescue analgesia was significantly lower in the S2 group (χ^2^ *p*=0.01) (Table 5). Group S1 and S2 patients required first rescue analgesic after 4hours into the postoperative period, while most of the Group P patients required rescue analgesic within 2hours post-extubation.

## Discussion

The statistical analysis shows that patients in the three groups were comparable with regard to age, sex and ASA physical status; however, they differed with regard to body weight. The mean body weight was higher in group P compared to Groups S1 and S2. This is taken into consideration in the statistical analysis of haemodynamic parameters by adjusting for potential interactions between covariates like age, sex, weight, duration of anaesthesia, volume of propofol and sufentanil, and baseline haemodynamic parameters.

No statistically significant difference was seen in the haemodynamic parameters across the three groups of patients in the perioperative period. Neither intubation stimulus, nor surgical incision, nor peritoneal insufflation with CO_2_ had any influence on the haemodynamic parameters in all groups. This may be attributed to the bolus of sufentanil (1μg kg^−1^) given at induction. Our study confirms that synergistic pharmacodynamic interactions between propofol and sufentanil block responses to laparoscopic surgery. Similar observations were made in other surgical procedures^8^,^9^. Lysakowski et al^10^ studied the effects of fentanyl and its congeners on loss of consciousness during propofol induction of anaesthesia and concluded that analgesic concentrations of sufentanil enhance the anaesthetic effect of propofol and provide haemodynamic stability.

We observed that fewer Group S2 patients required additional boluses of sufentanil to maintain adequate depth of anaesthesia. The lesser requirement of boluses is a consequence of haemodynamic stability which in turn can be attributed to higher concentration of sufentanil (2 μg ml^−1^) used in Group S2. The bolus requirement was more frequent in the Groups P and S1, which could have contributed to the stability seen in haemodynamic parameters in these groups. The mean decrease in the heart rate, systolic and diastolic blood pressures in perioperative period in the study groups was within a clinically acceptable range. Monk et al^1^ reported similar findings of intraoperative haemodynamic stability with sufentanil compared to other opioids. Elisabeth Hentgen et al^11^ combined propofol and sufentanil in TIVA for thyroid surgery and observed that increasing target concentration of sufentanil provided better haemodynamic stability.

The sufentanil induction dose and propofol infusion rates used in our study were chosen based on dosage regimen suggested in earlier publications.^1^,^2^ Previous studies showed that increasing concentrations of sufentanil reduce the volume of propofol consumed during the surgery.^3^,^11^ Although our study did not find a statistical difference in the total consumption of propofol, clinically, there was lesser consumption in Group S2 patients (Table 3). This marginal decrease could be due to lower body weight of the patients in this group or due to the higher concentration of sufentanil used. Alarger sample size might be able to decisively answer this question.

Recovery time was significantly prolonged (*p*=0.001) in Group S2 compared to the other two Groups. This delayed recovery in Group S2 patients, can be attributed to requirement of more than one sufentanil bolus in 15% of patients in addition to the higher concentration of sufentanil (2 μg ml^−1^). No significant difference was observed in Group S1 where 50% patients required one sufentanil bolus in addition to 1 μg ml^−1^ concentration of sufentanil, compared to Group P where about 20% patients needed multiple boluses (Table 4). Similar conclusions were drawn by Elisabeth Hentgen et al^11^ in a previous study that combined increasing target concentrations of sufentanil with propofol in TIVA for thyroid surgery. Our findings in laparoscopic cholecystectomy confirm the conclusions reached by earlier studies of sufentanil in other surgical procedures. It is important to note that the observed 7-minute prolongation of recovery time was clinically insignificant in our setting where the patients undergoing laparoscopic cholecystectomy get discharged from the hospital on the firstpostoperative day.

Postoperative analgesia was better in Group S2 (χ^2^ *p*=0.01) compared with the other two groups. Patients in Group S2 had excellent 24-hour postoperative analgesia (VAS score less than 3) with least requirement of rescue analgesia. Group S1 patients had good early postoperative analgesia with greater rescue analgesic requirement in the later postoperative period (more than 4 hours postoperatively). In patients who received plain propofol (Group P) there was a greater requirement of intraoperative sufentanil boluses and highest rescue analgesic requirement in the early post-operative period compared to the other two groups. Derrode et al^6^ compared effects of target-controlled infusions of sufentanil and remifentanil administered along with propofol on recovery and postoperative analgesia after major abdominal surgery. They observed that quality of postoperative analgesia depends on the opioid infused during surgery and concluded that intra-operative use of sufentanil was very effective in providing excellent 24-hour postoperative analgesia. Our study also suggests that sufentanil at a concentration of 2 μg ml^−1^ provides better quality of intraoperative and postoperative analgesia in laparoscopic cholecystectomy.

In summary, sufentanil mixed in propofol for TIVA in laparoscopic cholecystectomy gives adequate depth of anaesthesia, stable haemodynamics, good postoperative analgesia, and a slightly prolonged recovery time.

Sufentanil is an effective adjuvant in TIVA for laparoscopic cholecystectomy. This study concludes that both concentrations of sufentanil achieve the goals of stable haemodynamics without a clinically significant prolongation of recovery time. However, 2 μg ml^−1^ concentration of sufentanil added to propofol provided greater perioperative haemodynamic stability with lesser requirement for additional boluses and excellent post-operative analgesia. The influence of sufentanil on recovery times needs further clinical trials with larger sample sizes.
